# Hematopoietic stem cell gene editing rescues B-cell development in X-linked agammaglobulinemia

**DOI:** 10.1016/j.jaci.2024.03.003

**Published:** 2024-07

**Authors:** Sameer Bahal, Marta Zinicola, Shefta E Moula, Thomas E. Whittaker, Andrea Schejtman, Asma Naseem, Elena Blanco, Winston Vetharoy, Yi-Ting Hu, Rajeev Rai, Eduardo Gomez-Castaneda, Catarina Cunha-Santos, Siobhan O. Burns, Emma C. Morris, Claire Booth, Giandomenico Turchiano, Alessia Cavazza, Adrian J. Thrasher, Giorgia Santilli

**Affiliations:** a Infection, Immunity and Inflammation Research and Teaching Department, University College London Great Ormond Street Institute of Child Health, London, United Kingdom; b University College London Institute of Immunity and Transplantation, London, United Kingdom; c Department of Immunology, Royal Free London National Health Service Foundation Trust, London, United Kingdom; d Great Ormond Street Hospital, National Health Service Foundation Trust, London, United Kingdom

**Keywords:** Agammaglobulinemia, B cell, gene editing, hematopoietic stem and progenitor cells

## Abstract

**Background:**

X-linked agammaglobulinemia (XLA) is an inborn error of immunity that renders boys susceptible to life-threatening infections due to loss of mature B cells and circulating immunoglobulins. It is caused by defects in the gene encoding the Bruton tyrosine kinase (BTK) that mediates the maturation of B cells in the bone marrow and their activation in the periphery. This paper reports on a gene editing protocol to achieve “knock-in” of a therapeutic BTK cassette in hematopoietic stem and progenitor cells (HSPCs) as a treatment for XLA.

**Methods:**

To rescue BTK expression, this study employed a clustered regularly interspaced short palindromic repeats/CRISPR-associated protein 9 system that creates a DNA double-strand break in an early exon of the *BTK* locus and an adeno-associated virus 6 virus that carries the donor template for homology-directed repair. The investigators evaluated the efficacy of the gene editing approach in HSPCs from patients with XLA that were cultured *in vitro* under B-cell differentiation conditions or that were transplanted in immunodeficient mice to study B-cell output *in vivo*.

**Results:**

A (feeder-free) B-cell differentiation protocol was successfully applied to blood-mobilized HSPCs to reproduce *in vitro* the defects in B-cell maturation observed in patients with XLA. Using this system, the investigators could show the rescue of B-cell maturation by gene editing. Transplantation of edited XLA HSPCs into immunodeficient mice led to restoration of the human B-cell lineage compartment in the bone marrow and immunoglobulin production in the periphery.

**Conclusions:**

Gene editing efficiencies above 30% could be consistently achieved in human HSPCs. Given the potential selective advantage of corrected cells, as suggested by skewed X-linked inactivation in carrier females and by competitive repopulating experiments in mouse models, this work demonstrates the potential of this strategy as a future definitive therapy for XLA.

## Graphical abstract

**Figure:**
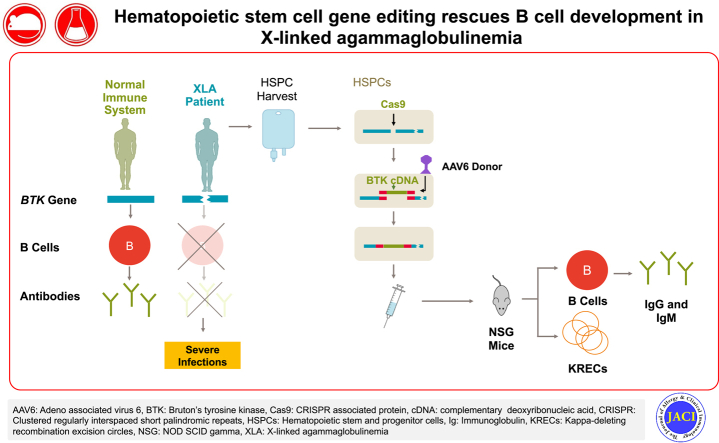

Antibody deficiencies are the most prevalent cause of inborn errors of immunity accounting for >50% of cases. Among these, X-linked agammaglobulinemia (XLA) represents one of the most severe forms, affecting around 1:100,000 newborn males.^1^ XLA arises from mutations in the gene encoding BTK, a nonreceptor tyrosine kinase, belonging to the Tec family, that promotes the maturation of B cells in the bone marrow and their activation in the periphery.^2^^,^^3^ At the pro–B-cell stage, the expression of BLNK, Igα, Igβ, and the RAG1- and RAG2-mediated production of Igμ lead to cell surface expression of the pre–B-cell receptor. This complex generates survival signals in pre–B cells via BTK. Consequently, defects in BTK cause a cessation of B-cell development and usually a complete antibody deficiency. Patients with XLA suffer frequent and severe bacterial infections from around 6 months of age as waning passive maternal humoral immunity results in agammaglobulinemia. The mainstay of infection prevention is immunoglobulin replacement therapy (IgRT), a life-long therapy requiring regular intravenous or subcutaneous administration that also places a substantial economic burden on health systems, and it is not consistently available in all countries. Despite IgRT, severe and chronic breakthrough infections occur. Most commonly, these are sinopulmonary infections, otitis media, and diarrheal illnesses.^4^ Indeed, current IgRT does not rescue other roles of B cells such as regulation of dendritic cells and costimulation of T-cell lymphocytes as well as defects in the BTK–Toll-like receptor signaling in other lineages.^5^ Additionally, the quality of life of patients with XLA is significantly worse when compared to healthy individuals, due to both treatment and disease burden.^6^ Finally, patients with XLA suffer a higher risk of gastrointestinal tract malignancy and lymphoma.^7^

The only definitive cure for XLA is hematopoietic stem cell (HSC) transplantation, which is not routinely offered to patients who are stable on IgRT, given the risks associated with graft versus host disease and the conditioning regimen. A recent report on a patient with XLA who underwent transplantation showed clearance of norovirus infection prior to humoral reconstitution, confirming a nonhumoral component in XLA, and highlighting the importance of broad cellular correction.^8^ Genetic correction of autologous HSCs can overcome the risks associated with allogeneic stem cell transplantation.^9^ Optimized lentiviral vectors containing B-cell–specific promoters have highlighted the importance of restoring physiological levels of BTK^10^, ^11^, ^12^, ^13^; low expression impairs humoral reconstitution,^10^^,^^12^ while high expression could potentially be oncogenic.^12^^,^^14^

The advent of gene editing has opened new avenues of treatment for monogenic disorders such as XLA, where physiological expression is critical. Gene editing uses programmable nucleases to create precise, site-specific double-strand breaks (DSBs) within the genomic DNA, enabling targeted modifications during the subsequent DNA repair process. When the cell is provided with a donor template containing regions of homology to either side of the DSB, homology-directed repair (HDR) can allow for the specific integration of the whole therapeutic transgene/cDNA in a one-size-fits-all approach. Clustered regularly interspaced short palindromic repeats/CRISPR-associated protein 9 (CRISPR/Cas9) is the most widely used designer nuclease given its adaptability, and reports of gene editing strategies using this system for HSC gene editing are abundant.^15^ Stemming from the original report on CRISPR/Cas9 are relatively novel strategies to address point mutations without the need to create DSBs such as base or prime editors.^16^^,^^17^

For XLA, however, precise correction of single individual mutations using gene/base editing systems is not a viable clinical option given that there are over 600 different mutated sites in *BTK*.^18^ HDR-mediated targeted insertion of the *BTK* coding sequence adjacent to native regulatory sequences can address almost all the mutations in *BTK* and potentially achieve physiological levels of BTK. Normal levels of BTK were obtained with an adenovirus/adeno-associated virus (AAV) hybrid vector targeting genomic insertion in exon 6 of *BTK*, but efficiency was low.^19^ A CRISPR/Cas9-based one-size-fits-all approach has been tested in BTK-deficient cell lines and revealed the potential importance of intronic regions to sustain physiological levels of BTK.^20^

In this study we report on a gene editing strategy designed to achieve normally regulated levels of BTK in relevant cells from patients with XLA.

While the relatively low frequency of HDR-mediated correction of primitive, repopulating HSPCs hampers the clinical translation of these approaches for most disorders, XLA would be an ideal candidate for a gene editing approach, as suggested by the presence of nonrandom X-linked inactivation in XLA female carriers as well as studies on murine models.^21^^,^^22^ Indeed, B-cell development could reliably be restored, in a mouse model of XLA, with transplantation of mixtures of cells containing as few as 5% of healthy, wild-type (WT) HSPCs.^22^ Here we report an editing efficiency of >30% in patient-derived HSPCs and the rescue of B-cell development and antibody production *in vitro* and *in vivo*.

## Methods

### Cell cultures

The Burkitt lymphoma cell line DG75 and the T-cell leukemic cell line, Jurkat, were cultured in RPMI media supplemented with 10% FBS and 1% penicillin streptomycin (Gibco, Thermo Fisher Scientific, Waltham, Mass). CD34^+^ hematopoietic stem and progenitor cells (HSPCs) from healthy donors (AllCells, Alameda, Calif) and patients with XLA were isolated as described in the Methods in this article’s Online Repository (available at www.jacionline.org) and cultured in StemSpan Serum-Free Expansion Medium II, (cat no: 09605; STEMCELL Technologies, Vancouver, British Columbia, Canada) supplemented with penicillin-streptomycin (1%), 20 ng/mL IL-6, 100 mg/mL Fms-like tyrosine kinase 3 ligand (Flt3L), 100 ng/mL Stem Cell Factor (SCF), 60 ng/mL IL-3, 20 ng/mL Thrombopoietin (TPO) (all cytokines from PeproTech, London, United Kingdon), UM729 (500 nmol/L, STEMCELL Technologies), and StemReginin 1 (1 μmol/L; Cambridge Bioscience Ltd, Cambridge, United Kingdom). For semisolid cultures, 1250 cells in 500 μL of media were added to a 2.5-mL aliquot of methylcellulose, StemMACS HSC-CFU (Miltenyi Biotec, Bergisch Gladbach, Germany). After vortexing, 1 mL was pipetted into wells of a 12-well plate. Erythroid and myeloid colonies were counted after 10 days.

### B-cell differentiation culture

CD34^+^ cells were cultured in a specialized B-cell differentiation media comprising Iscove modified Dulbecco medium supplemented with 10% FBS, minimum essential medium nonessential amino acids (1%), insulin (1 μg/mL), transferrin (2.5 μg/mL), reduced glutathione (1 μg/mL), and penicillin streptomycin (1%). For the first 2 weeks, SCF (25 ng/mL) and Flt3L (25 ng/mL) were added. In addition, IL-6 (25 ng/mL) was added in the first week and replaced with IL-7 (20 ng/mL) for the second week. Cells were cultured in a plate coated with ICAM1-Fc (cat no: 552906; BioLegend, San Diego, Calif) from week 2 onward and in cytokine-free media from week 3 onward.

### T-cell cocultures

Naïve CD4^+^ cells were isolated from healthy donor PBMCs using a MACS kit (Miltenyi Biotec). Then 50,000 cells from day 30 of the B-cell differentiation cultures were mixed with CD4^+^ cells in a 1:1 ratio in the presence of staphylococcal enterotoxin B (SEB) (50 ng/mL; Sigma-Aldrich, Gillingham, Dorset, United Kingdom). After 7 days, the supernatants were analyzed by ELISA to determine IgG (cat no: BMS2091; Invitrogen, Thermo Fisher Scientific) and IgM (cat no: BMS2098, Invitrogen) content, following manufacturer’s recommendations. T cells were analyzed for activation markers by flow cytometry.

### Cloning of donor templates and AAV6 preparation

All HDR donor templates were cloned into the pAAV vector plasmid containing AAV2-specific inverted terminal repeats. The donor templates contain left and right homology arms of 813 and 585 bp in length, respectively, and a bovine growth hormone poly A signal. The AAV6-co-BTK contains a codon optimized BTK cDNA that was synthesized by Invitrogen GeneArt and, where indicated, a canonical Kozak sequence with or without a 300-bp region of the *BTK* intron 18. The AAV6-green fluorescent protein (GFP) construct contains a PGK-GFP reporter cassette. AAV6s were produced by transient transfection of HEK293T cells with the transfer and the pDGM6 Rep/Cap helper plasmids (as described in the Methods in this article’s Online Repository at www.jacionline.org).

### Editing cell lines and HSPCs

Jurkat T cells and DG75 cells were electroporated using the Neon Transfection kit (Thermo Fisher Scientific) at 1600 volts for 3 pulses lasting 10 milliseconds each. The ribonucleoprotein (RNP) complex was made by combining High Fidelity Cas9 protein (cat no: 1081061; Integrated DNA Technologies, Coralville, Iowa) with chemically modified guide RNAs (gRNAs) (Synthego Corporation, Redwood City, Calif) targeting *BTK* exon 2 at a molar ratio of 1:2 (Cas9: gRNA). Cells were then placed in RPMI with 10% FBS and 1% penicillin streptomycin and AAV6 donor virus was added 15 minutes later at a multiplicity of infection (MOI) of 20,000. Cells were left for 1 week to recover before deriving clones. HSPCs were cultured for 2 days before being electroporated with the RNP complex (described above) using the MaxCyte GT platform with the HSC-3 program (MaxCyte Inc, Rockville, Md). Cells were then cultured in the fully supplemented StemSpam medium. AAV6 donors, at the indicated MOIs, were added 15 minutes later.

### Flow cytometry analysis

For the flow cytometry analysis, 100,000 HSPCs undergoing B-cell differentiation were stained with the following antibodies: CD19–APC/Cy7 (clone HIB19, cat no: 302218), CD10-APC (cloneHI10a, cat no: 312210), and IgM-Alexa Flour 700 (clone MHM-88, cat no: 314538). T cells from the coculture system were stained with CD4–fluorescein isothiocyanate (clone RPA-T4), programmed cell death protein 1–APC (clone A17188B), and CXCR5-BV421 (clone RF8B2). Bone marrow cells were stained with a B-cell panel: CD19–PE (clone HIB19, cat no: 302208), human CD45 antibody (hCD45)–PerCP/Cy5.5 (clone HI30, cat no: 304028), CD34-PE Cy7 (clone 581, cat no: 343516), CD10-APC (clone HI10a, cat no: 312210), and IgM-APC Cy7.0 (clone MHM-88, cat no: 314520), or they were stained with a CD34 cell panel: hCD45-PerCP/Cy5.5 (clone HI30, cat no: 304028), CD34-BV421 (clone 561, cat no: 343610), CD45RA-APC (clone HI100, cat no: 304112), CD38-APC Cy7.0 (clone HIT2, cat no: 303534), and CD90-PE Cy7 (clone 5E10, cat no: 328124). Blood cells were stained with CD19-PE (clone HIB19, cat no: 302208) and hCD45-PerCP/Cy5.5 (clone HI30, cat no: 304028). All antibodies were from BioLegend. DAPI (4′,6-diamidino-2-phenylindole) at a concentration of 3 μmol/L was used to exclude dead cells. Data acquisition was performed with an LSR II flow cytometer (BD Biosciences, Franklin Lakes, NJ) and analyses with FlowJo software, version 10 (BD Biosciences).

### Droplet digital PCR

The frequency of integrated BTK (copies per diploid genome: 2× copies of co-BTK/copies of albumin) or κ-deleting recombination excision circles (KRECs) production (copies per diploid genome: 2× copies of KRECs/copies of *TRAC*) were quantified by droplet digital PCR (ddPCR). The KRECs’ detection methodology was adapted from previous works.^23^^,^^24^ Reactions were assembled with 11 μmol/L 2× ddPCR Supermix for Probes (no dUTP) (Bio-Rad Laboratories, Hercules, Calif), 0.8 μL of each 10 μmol/L primer, 0.4 μL of each 10 μmol/L probe, 2 μL sample DNA, and nuclease-free water to 22 μL. Droplets were generated using a QX200 AutoDG, amplified with a C1000 Touch Thermal Cycler, and analyzed with a QX200 droplet reader (Bio-Rad). A list of primers and probes and cycling conditions is shown in Tables E1 and E2 in this article’s Online Repository (available at www.jacionline.org).

### Xenotransplantation studies

Male and female NOD.Cg-Prkdc^scid^ Il2rg^tm1Wjl^/SzJ (NSG) mice were obtained from Charles River Laboratories (Wilmington, Mass) and were housed in ventilated cages containing sterile bedding, food, and water in a temperature- and humidity-controlled environment. Mice were sublethally irradiated with 3 Gy using an IBL 437 C machine (CIS Bio International, Codolet, France). The following day, 2 million HSPCs were transplanted into each mouse via a tail vein injection. After 15 weeks, bone marrow and blood were harvested.

Plasma was separated from blood samples by centrifugation at 2000*g* for 40 minutes and used for ELISA studies.

### Genotoxicity studies

DNA was extracted from 1,500,000 XLA1 and XLA2 HSPCs 4 days after they had been electroporated with Cas9/gRNA only. Chromosomal aberrations analysis by single targeted linker-mediated PCR sequencing (CAST-seq) was performed as described previously^25^ with the following specific primers: BTK Bait GTGCACGGTCAAGAGAAAC; BTK Decoy GTTCACCTGTGTGCTGTTG; and BTK Nested GACTGGAGTTCAGACGTGTGCTCTTCCGATCTCGCTTCTTGAAGTTTAGAGGTGATG. DNA from untreated XLA1 and XLA2 samples was used as a control. Predicted off-target effects were selected using the COSMID online software.^26^ These sites were PCR amplified and pooled prior to purification using a Monarch PCR and DNA Cleanup Kit (cat no: T1030L, New England Biolabs, Ipswich, Mass). A list of primers is in Table E1. End repair was performed with the NEBNext Ultra II End Repair/dA-Tailing Module (cat no: E7546S, New England Biolabs). Linker ligation was performed with the NEBNext Ultra II Ligation Module (cat no: E7595S, New England Biolabs). Then 3 μL of USER Enzyme (cat no: M5505S, New England Biolabs) was added to each sample and incubated at 30°C for 15 minutes. Samples were then barcoded using a NEBNext Kit (cat no: E6440S, New England Biolabs). An Ampure XP Kit (cat no: A63881; Beckman Coulter, Pasadena, Calif) was used to purify the products post barcoding. Next-generation sequencing was performed on samples using the MiSeq System (Illumina, San Diego, Calif) with the MiSeq Reagent Kit v3 (600-cycle kit) (Illumina). Insertions and deletions (INDELs) were analyzed using CRISPResso2^27^ with a quantification window size of 10 bases and ignoring the substitutions.

### Statistical analysis

Statistical analysis was performed with GraphPad Prism, version 9 (GraphPad Software, San Diego, Calif). For comparisons between 2 groups, a 2-tailed Student *t* test was used. For comparison among >2 groups, we used ANOVA followed by Tukey’s multiple comparisons.

For the experiments shown in Fig 6, *A*, a 1-tailed *Z-*test was used to compare the INDEL proportions in the treated versus untreated samples correcting for the SD value obtained in the untreated readouts, to account for measurements’ variability.

## Results

### CRISPR/Cas9-mediated targeted insertion of a BTK optimal cassette in cell lines rescues physiological BTK expression

**Fig 1:**
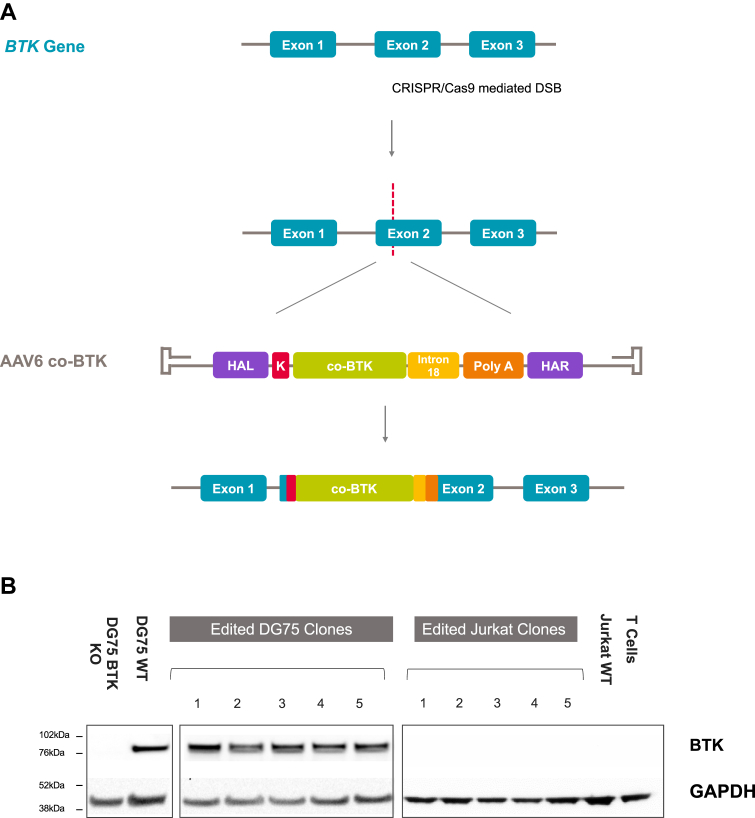
Optimization of a gene editing platform for XLA. **A,** Schematic of the gene editing strategy to knock in a BTK coding sequence into exon 2 of the native locus using the CRISPR/Cas9 system and an AAV6 to deliver the HDR donor template. The HDR donor template consists of a codon optimized version of the BTK coding sequence (*co-BTK*) with a canonical GCCACCATGG Kozak sequence (*K*), a 300 bp region of intron 18 (*I18*) and a βgrowth hormone polyadenylation signal (*poly A*), flanked by the homology arms left (*HAL*) and right (*HAR*). **B,** Western blot analysis of BTK expression in DG75 and Jurkat T-cell clones. DG75 and Jurkat T cells were electroporated with the Cas9/gRNA RNP and transduced with the AAV6 vector containing the co-BTK cassette before clones were derived. The GAPDH housekeeping protein is used as a loading control.

**Fig E1:**
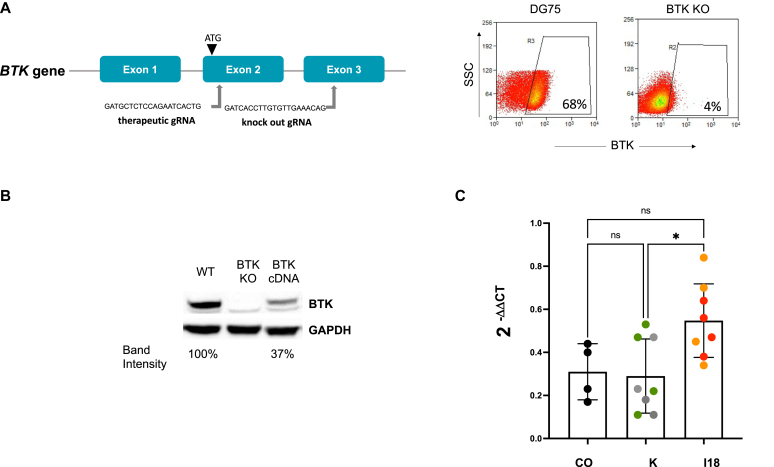
Optimization of the AAV6–co-BTK donor cassette in DG75 BTK KO cells. **A,** A representation of the *BTK* locus is shown, including the sequence of the therapeutic gRNA targeting exon 2 and the gRNA used to knockout *BTK* by mutating exon 3. *Arrows* show the approximate cut site of both gRNAs (*left*). DG75 cells were edited with a Cas9/gRNA RNP targeting exon 3 of the *BTK* locus before deriving a clonal KO population (DG75 KO). Flow cytometry analysis of BTK expression in WT and KO DG75 cells (*right*). **B,** DG75 BTK KO cells were edited with a Cas9/gRNA RNP complex targeting exon 2 and transduced with an AAV6 HDR donor template containing a codon optimized BTK coding sequence, before deriving clonal populations. Western blot analysis of BTK levels in WT, KO, and a BTK “knocked in” clone. **C,** Real-time quantitative PCR evaluation of BTK mRNA expression in clones knocked in with a BTK codon-optimized coding sequence (*CO*; *black dots*) and a BTK codon-optimized coding sequence containing a canonical Kozak sequence alone (*K*; each clone shown with different color) or with a 300-bp region from I18 (*I18*; each clone with different color). The 2^−ΔΔCT^ values [Delta Ct single clones (Ct coBTK-Ct GAPDH)-deltaCt WT (Ct wtBTK-Ct GAPDH)] are shown. Of note, primers to amplify the WT and codon optimized BTK differ, so conclusions on the relative amount of BTK versus WT cannot be drawn. (Shown is the mean ± SD; ∗*P* < .005 1-way ANOVA with Tukey’s multiple comparison.) *NS*, Not significant; *SSC*, side scatter.

To accomplish integration of a *BTK* coding sequence under its native regulatory elements, we used a CRISPR/Cas9 system delivered as a RNP complex to induce a DSB in exon 2 of the *BTK* locus, and an AAV6 vector to deliver an optimal donor template for HDR. The donor template contains a codon-optimized BTK cDNA (exons 2-19) with a canonical Kozak sequence followed by 300 bp of *BTK* intron 18 and a polyadenylation signal from the bovine growth hormone, flanked by regions of homology to the DSB (Fig 1, *A*). We have selected this optimal donor design after evaluating different donor conformations in an in-house generated BTK knockout (KO) DG75 B-cell line (see Fig E1, *A* in this article’s Online Repository at www.jacionline.org). An initial construct with a codon optimized BTK cDNA only resulted in low levels of BTK expression, in line with previous reports^20^ (Fig E1, *B*), and prompted us to test 2 alternative donor designs containing a canonical Kozak sequence without or with a 300-bp region of intron 18. This region contains putative binding sites for proteins involved in RNA processing either as part of the spliceosome complex or independently.^28^ DG75 clones reconstituted with the latter (optimal donor template) showed the highest BTK mRNA levels (Fig E1, *C*), although protein levels were similar to those observed in clones carrying the intronless donor template (data not shown). Importantly, when protein levels were evaluated in both T- and B-cell lines (Fig 1, *B*), clones showed levels of BTK comparable to WT in B cells, but no ectopic expression in Jurkat T cells, reflecting a physiological pattern of BTK expression. Primary human T cells were used to confirm that BTK is not expressed in this lineage as previously reported.^29^^,^^30^

### Gene editing of human XLA CD34^+^ HSPCs rescues the B-cell developmental defects *in vitro*

**Fig 2:**
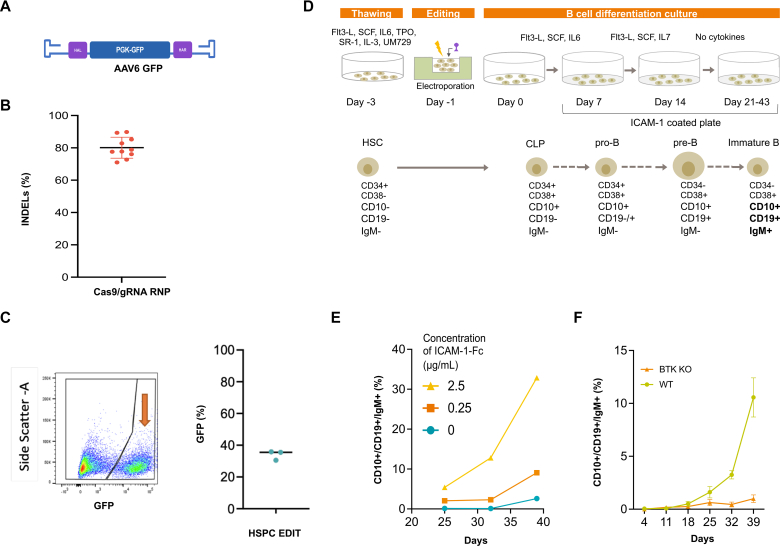
Gene editing efficiency in HSPCs and optimization of a B-cell differentiation protocol. **A,** Schematic of the donor construct (AAV6-GFP) used to knock in a PGK-GFP cassette in the *BTK* locus creating BTK KO HSPCs. **B,** Cutting efficiency of the Cas9/gRNA RNP complex targeting *BTK* exon 2 in multiple HSPC donors (n = 10) as determined by TIDE analysis. **C,** Frequency of HDR-mediated targeted insertion of PGK-GFP in the *BTK* locus. HSPCs (n = 3) were electroporated with Cas9/gRNA RNP, transduced with the AAV6-GFP donor vector, and analyzed by flow cytometry for high GFP expression as a measure of targeted integration. Representative flow cytometry plot showing GFP expression out of live cells (*left*) with recapitulating plot (*right*). **D,** Overview of the HSPC editing and B-cell differentiation protocol adapted from Kraus et al.^33^**E,** Frequency of immature B cells (defined as CD19^+^/CD10^+^/IgM^+^) emerging from WT HSPCs at different days of the B-cell differentiation protocol when using increasing concentrations of ICAM1-Fc coating agent. **F,** Development of CD10^+^/CD19^+^/IgM^+^ immature B cells from GFP^+^ BTK KO HSPCs compared with WT HSPCs, which had been placed in B-cell differentiation culture (n = 3; mean ±SD). *CLP*, Common lymphoid progenitor; *SCF*, stem cell factor; *SR1*, StemRegenin 1; *UM729*, Methyl 4-((3-(piperidin-1-yl) propyl) amino)-9H-pyrimido[4,5-b] indole-7-carboxylate.

We proceeded to test the gene editing strategy on HSPCs from healthy donors using an AAV6 vector to target the insertion of a PGK-GFP reporter cassette into the *BTK* locus (Fig 2, *A*). Cells were nucleofected with the high-fidelity Cas9/gRNA RNP and transduced with an AAV6-GFP. INDELs at the cut site were determined to be around 80% via TIDE (Tracking of Indels by DEcomposition) analysis (Fig 2, *B*).^31^ At day 4 post editing, the proportion of cells expressing high levels of GFP, suggestive of an integration event,^32^ was >30% in HSPCs across donors (Fig 2, *C*).

With the aim of establishing a functional readout for the gene editing strategy, we adapted a previously described feeder-free B-cell differentiation culture system^33^ to derive immature B cells (Fig 2, *D*). Different concentrations of ICAM1-Fc, used to coat culture wells during B cells replating to mimic stroma, affected the frequency of immature B cells and could potentially mask the difference between XLA and WT samples (Fig 2, *E*). Therefore, we decided to use a low concentration of 0.25 μg/mL.

HSPCs with defects in *BTK* show a block in the maturation and lack of pre-B and immature B cells. We used WT HSPCs where the PGK-GFP cassette was “knocked-into” the *BTK* locus as control GFP-positive–BTK KO cells. When GFP-positive-BTK KO cells underwent B-cell differentiation in this system, we observed a diminished output of CD10^+^/CD19^+^/IgM^+^ immature B cells compared to WT HSPCs (0.99 ± 0.36% vs 10.56 ± 1.85%, respectively) (Fig 2, *F*), confirming the suitability of this system as a readout of the functional efficacy of our gene editing approach in patient samples.

**Fig 3:**
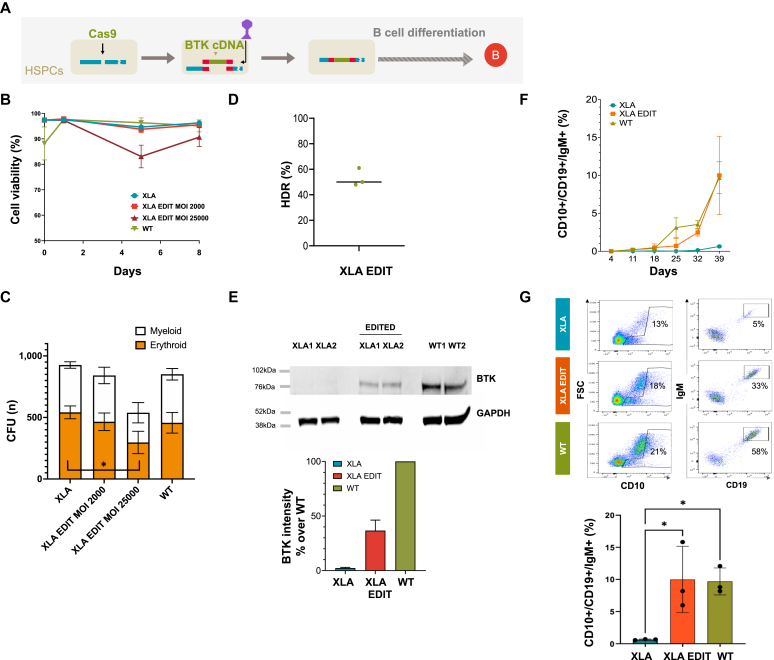
Gene editing of XLA HSPCs rescues the B-cell developmental block. **A,** Rescue of the XLA phenotype after gene editing: experimental overview. XLA HSPCs were electroporated with the Cas9/gRNA RNP (*Cas9*) and transduced with the AAV6 co-BTK donor (*BTK cDNA*) vector before undergoing B-cell differentiation. **B,** Cell viability over time of WT HSPCs, nonedited XLA HSPCs and XLA HSPCs edited with high-dose (XLA EDIT MOI: 25000) and low-dose (XLA EDIT MOI: 2000) AAV6 as assessed by trypan blue analysis. **C,** Frequency of myeloid (*white*) versus erythroid (*orange*) colonies derived from methylcellulose cultures of the samples as in **B**. **D,** Frequency of HDR-mediated targeted insertion of the BTK cassette in XLA HSPCs (n = 3) edited using an AAV6 (MOI: 2000), as assessed by ddPCR. **E,** Western blot analysis of BTK protein expression in WT, XLA nonedited and edited samples at day 25 of B-cell differentiation (*upper*). WT samples are used as control for protein expression. GAPDH is used as loading control. Densitometric analysis is shown (*lower*), as a proportion of WT (100%) (data are shown as mean ± SD; n = 3). **F,** Proportion of immature B cells (CD10^+^/CD19^+^/IgM^+^) emerging over time from the 3 edited XLA HSPCs undergoing B-cell differentiation along with nonedited XLA and WT. **G,** Representative flow cytometry plot of day 39 (*top*) with a summarizing histogram (*bottom*) (n = 3, mean ± SD, 1-way ANOVA followed by Tukey’s multiple comparisons; significance is indicated as ∗*P* < .05). *CFU*, Colony-forming unit.

**Fig E2:**
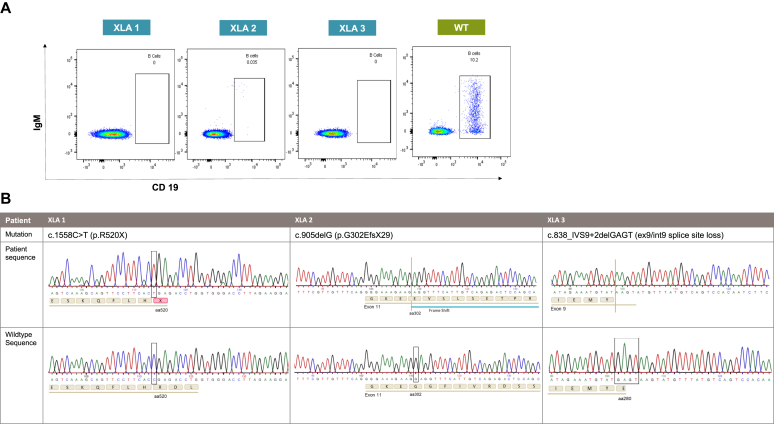
Confirmation of XLA phenotype in XLA HSPCs donors. **A,** Flow cytometry performed on peripheral blood from 3 XLA HSPCs donors showing staining for CD19 and IgM. **B,** Table showing the disease-causing mutations of each XLA donor with confirmatory Sanger sequencing traces comparing patient and WT reads.

**Fig E3:**
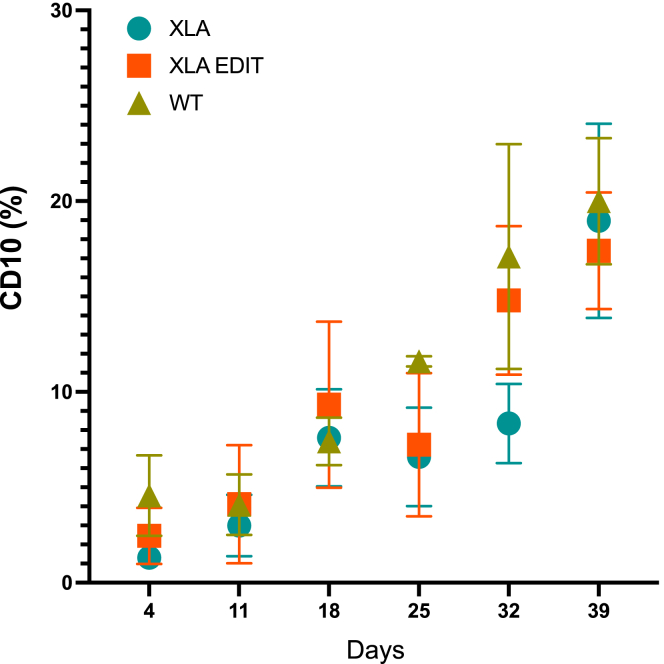
Similar proportion of CD10^+^ cells among XLA, XLA edit, and WT groups. Proportion of CD10^+^ cells emerging over time from WT, nonedited XLA, and edited XLA HSPCs undergoing B-cell differentiation (n = 3 different donors, mean ± SD, no statistical difference was found with repeated measures 2-way ANOVA followed by Tukey’s multiple comparisons).

We then obtained HSPCs from 3 patients with XLA with no circulating B cells (see Fig E2 in this article’s Online Repository at www.jacionline.org) to perform editing and *in vitro* B-cell differentiation (Fig 3, *A*). XLA HSPCs were edited using the AAV6–co-BTK optimal donor template at high (25000) and low (2000) MOI. We assessed the fitness of HSPCs after editing by evaluating cell viability (Fig 3, *B*) and colony forming unit potential (Fig 3, *C*). While cell viability remained unchanged among WT, XLA and gene edited XLA groups using a low dose of AAV6 (MOI 2000), the high dose group (MOI 25000) showed reduced viability over time and a reduced colony-forming unit potential with a significant decrease in erythroid colonies. Therefore, a low AAV6 MOI of 2000 was used in further experiments, after confirming >40% targeted integration rates by ddPCR (Fig 3, *D*). Analysis of protein expression by Western blot confirmed the presence of BTK in the edited samples undergoing B-cell differentiation (Fig 3, *E*, upper panel). Levels of BTK were proportional to the frequency of transgene knock-in (Fig 3, *E*, lower panel). Cells bearing the CD10^+^ marker (expressed at all stages of the B-cell maturation) expanded similarly among the groups (see Fig E3 in this article’s Online Repository at www.jacionline.org). The proportion of CD10^+^/CD19^+^/IgM^+^ immature B cells increased over time (Fig 3, *F*) and at day 39 reached similar levels in WT and XLA gene edited groups (9.7 ± 2.1% and 9.9 ± 5.2%, respectively) but remained <1% in the XLA group (Fig 3, *G*), demonstrating the capacity for gene editing to rescue B-cell maturation in XLA HSPCs.

### Restored T-cell activating potential and IgM/IgG production in edited XLA-patient B cells emerging from *in vitro* cultures

**Fig 4:**
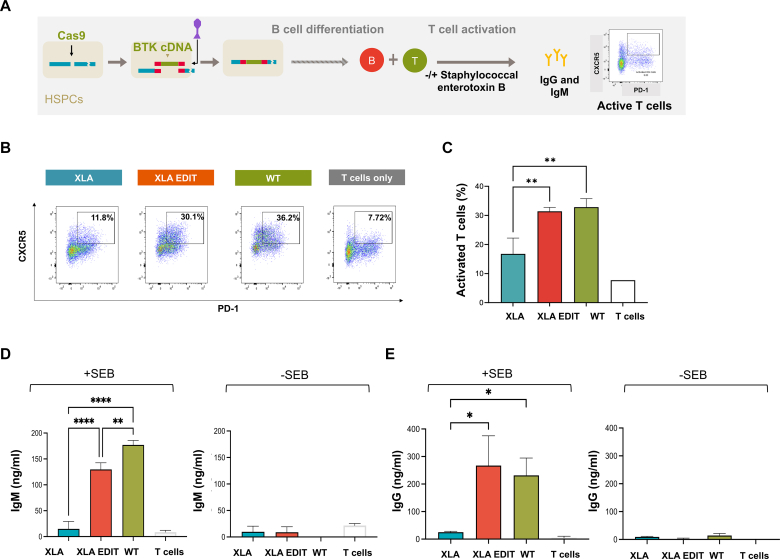
Gene editing restores B cells ability to activate T cells. **A,** Edited XLA HSPCs along with nonedited XLA and WT control groups were placed in B-cell differentiation culture. After 30 days, 50,000 cells from each condition were cocultured with primary CD4^+^ T cells, +/− SEB. After 7 days, activated follicular T cells were identified as the CXCR5^+^/programmed cell death protein 1 (*PD-1*) + population of CD4^+^ gated cells. Representative flow plots identifying activated T cells (**B**) and aggregate data (**C**). At the same time point, IgM **(D)** and IgG **(E)** concentrations in the supernatants were measured by ELISA (n = 3 different donors; shown is mean ± SD; 1-way ANOVA with Tukey’s multiple comparisons; ∗*P* < .05, ∗∗*P* < .01, ∗∗∗∗*P* < .0001).

We studied the functionality of gene edited cells in driving T-cell activation by coculturing emerging B cells with CD4^+^ T_H_ cells in the absence or presence of the superantigen SEB (Fig 4, *A*), promoting further maturation of B cells and immunoglobulin production.^34^

Coculture with samples from patients with XLA showed a low percentage of activated follicular T_H_ cells (CXCR5^+^/PD1^+^),^35^ indicating a relative failure of HSPC-derived B-cell development. In contrast, on editing of HSPCs from a patient with XLA, emerging B cells were able to activate T cells similarly to WT cells (as depicted in Fig 4, *B* and *C*). In response to T-cell stimulation, corrected XLA B cells produced IgM and IgG at levels comparable to healthy donor cells (Fig 4, *D* and *E*). As expected, IgM and IgG production from unstimulated cells was negligible.

### Edited XLA HSPCs reconstitute B-cell development in xenograft models

**Fig 5:**
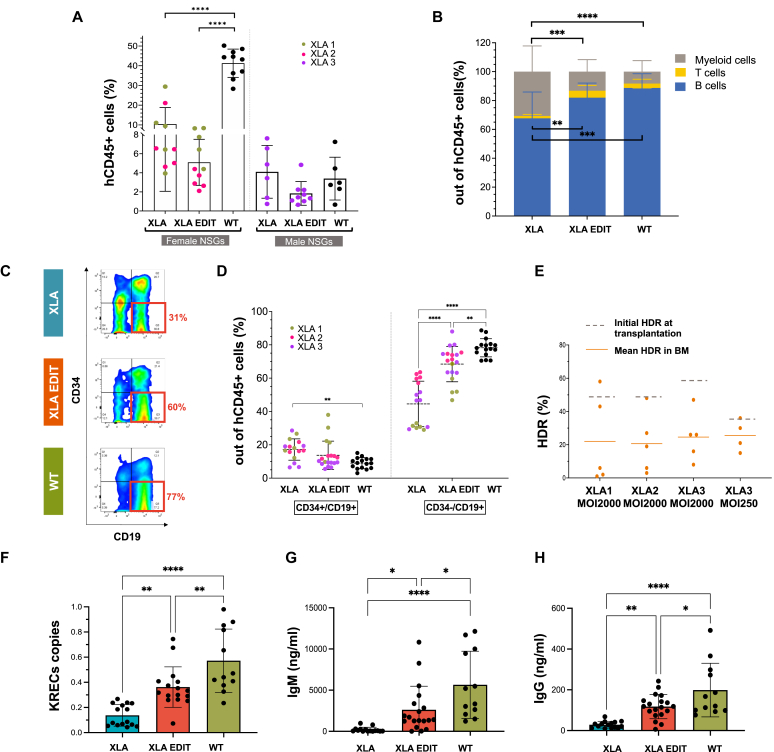
*In vivo* evaluation of edited XLA cells in xenotransplantation experiments. **A,** Edited XLA HSPCs (n = 10 mice for XLA donors 1 and 2; n = 9 mice for XLA donor 3) were transplanted into NSG mice in parallel with nonedited XLA HSPCs (n = 10 mice for XLA donors 1 and 2; n = 6 mice for XLA donor 3) and WT HSPCs (n = 10 and n = 6). Engraftment is shown as percentage of hCD45^+^ cells in the bone marrow (*BM*). **B,** Lineage composition of the human graft (CD33^+^ myeloid cells, CD19^+^ B cells and CD3^+^ T cells) in the bone marrow of mice transplanted with WT (n = 16), nonedited (n = 16), and edited (n = 19) XLA HSPCs. The total percentage of B-cell, T-cell, and myeloid lineages is set at 100%. **C and D,** Analysis of B cells subsets in the BM of transplanted mice. Representative flow cytometry plot (**C**) and aggregate frequency (**D**) of CD19^+^/CD34^−^ (cells at the pre–B-cell stage and onward) and CD19^+^/CD34^+^ (pro–B cells) in mice transplanted with WT HSPCs (n = 16) nonedited (n = 16) and edited XLA HSPCs (n = 19); each donor is depicted by a different color. **E,** Percentage of edited cells out of the total number of human cells (as assessed by ddPCR) in the BM of mice transplanted with XLA HSPCs edited using AAV6 co-BTK with MOI of 2000 for XLA1 and XLA2 and both MOIs of 2000 and 250 for XLA3. The HDR values in the HSPCs pretransplant are shown as a *dotted line.***F,** Average KREC copies per cell determined by ddPCR performed on BM DNA from mice that had been transplanted with WT HSPCs (n = 12), nonedited (n = 15) and edited (n = 16) XLA HSPCs. IgM **(G)** and IgG **(H)** concentrations (assessed by ELISA) in plasma samples of mice as in **F**. Data in **A, B, D, F-H** (mean ± SD); 1-way ANOVA followed by Tukey’s multiple comparisons: ∗*P* < .05, ∗∗*P* < .01, ∗∗∗*P* < .001, ∗∗∗∗*P* < .0001.

**Fig E4:**
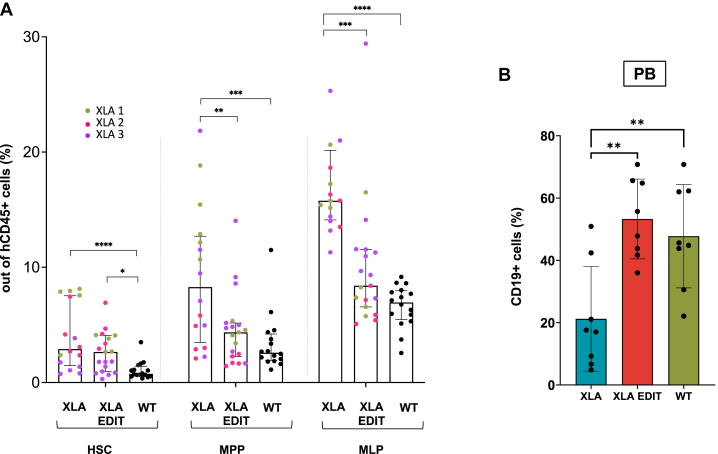
Gene editing restores a normal HSPC compartment in the bone marrow and normal levels of B cells in the blood of transplanted mice. **A,** Flow cytometry analysis of HSCs (CD34^+^/CD38^low^/CD45RA^−^/CD90^+^), multipotent progenitors (*MPPs*: CD34^+^/CD38^low^/CD45RA^−^/CD90^−^), and multi-lymphoid progenitors (*MLPs*: CD34^+^/CD38^low^/CD45RA^+^/CD90^−^) out of hCD45^+^ cells in the bone marrow of mice transplanted with WT (n = 16), nonedited (n = 16) and edited (n = 19) XLA HSPCs. **B,** Percentage of CD19^+^ B cells in the peripheral blood (*PB*) of mice transplanted with nonedited (n = 8) and edited (n = 8) XLA HSPCs. Mice transplanted with WT HSPCs (n = 8) were used as a control. (Shown is the mean ± SD; 1-way ANOVA with Tukey’s multiple comparisons; ∗*P* < .05, ∗∗*P* < .01, ∗∗∗*P* < .001, ∗∗∗∗*P* < .0001).

To assess the ability of edited HSPCs from patients with XLA to reconstitute the humoral system *in vivo*, we transplanted these cells into sublethally irradiated NSG male and female recipients. For 1 donor, we included an experimental group composed of mice transplanted with HSPCs edited with the AAV6 vector at a very low MOI (250) to determine whether lowering the MOI even further would improve engraftment rates. We investigated human cell engraftment and B-cell reconstitution in the bone marrow of transplanted animals. The overall engraftment was lower in male NSGs in keeping with previous data^36^ (Fig 5, *A*) and, in general, was low in the gene edited group as reported by others.^37^^,^^38^ We observed a higher percentage of myeloid cells at the expense of B cells in the bone marrow of mice transplanted with XLA cells in comparison to those transplanted with WT cells, with gene editing reversing this trend (Fig 5, *B*). As previously shown,^39^ the xenograft model did not faithfully recapitulate the phenotype observed in patients with XLA, but displayed an abrogated phenotype with CD34^−^/CD19^+^ cells (cells at the pre–B-cell stage and onward) present in the bone marrow of XLA transplanted mice. As expected, however, cells were found at a lower frequency than those in animals transplanted with WT cells and gene editing significantly rescued this phenotype (Fig 5, *C* and *D*). When looking at the HSPC subpopulations in the bone marrow, we found a higher percentage of multipotent progenitors and multilymphoid progenitors in the XLA group compared to the WT and gene edited group (see Fig E4, *A* in this article’s Online Repository at www.jacionline.org). The percentage of cells with targeted integration retrieved from transplanted mice is shown in Fig 5, *E* alongside pretransplant values.

Overall, the loss of corrected cells when using a high dosage of AAV6 (MOI: 2000) was greater than when using a very low dose (MOI: 250), so that, regardless of the starting point, the proportion of edited cells at 15 weeks post-transplantation did not differ between the edited groups. Next, we confirmed that immunoglobulin light chain rearrangement had taken place in the edited group by measuring KRECs in bone marrow DNA using ddPCR methodology (Fig 5, *F*).^23^ We then investigated immunoglobulin production. CD19^+^ B cells were found at comparable frequency in the peripheral blood of mice transplanted with WT and XLA edited cells and at lower frequency in those transplanted with XLA cells (Fig E4, *B*). More strikingly, analysis of plasma revealed a negligible production of IgM in XLA transplanted mice and a clear rescue of immunoglobulin secretion by gene editing (196.9 ± 296.3 ng/mL vs 2619 ± 2859 ng/mL; *P* < .05). A similar trend was also seen for IgG production (28.7 ± 16.5 ng/mL vs 117.8 ± 59.5 ng/mL; *P* < .01) (Fig 5, *G* and *H*).

### Safety profile of the gene editing approach

**Fig 6:**
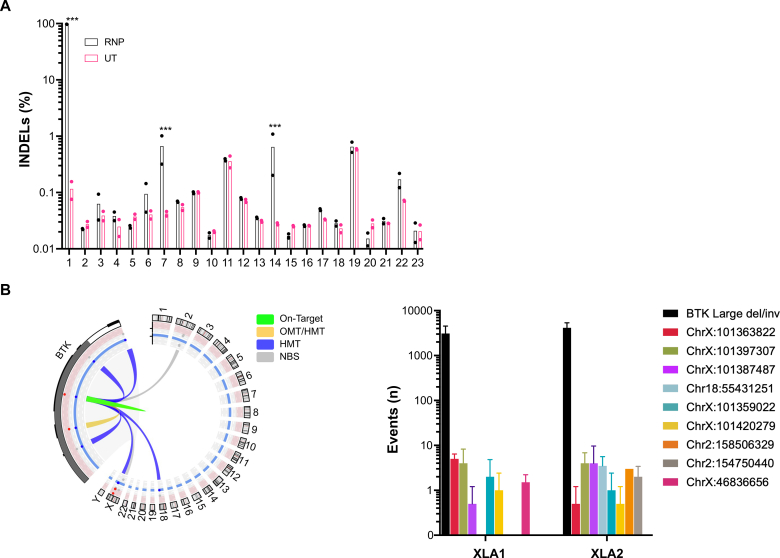
Genotoxicity analysis in edited XLA HSPCs. **A,** Targeted high-throughput sequencing of off-target sites predicted by COSMID in Cas9/gRNA RNP edited (*RNP*; n = 2) or untreated (*UT*; n = 2) XLA HSPCs. (∗∗∗*P* < .001 significant difference between Cas9/gRNA RNP and UT samples when analyzed with 1-tailed, 2-sample *Z* test of proportion) **B,** CAST-seq analysis of gross chromosomal aberrations in XLA HSPCs (n = 2). The Circos plot (*left*) shows a cluster of chromosomal rearrangements at the on-target site (*green*), at sites of homology to the *BTK* locus (a mix of *yellow* and *blue*), to chromosome X and 18 (*blue*), and at naturally occurring break sites (*NBS*; *gray*). Frequency of events found in the DNA from the 2 patients (*right*). *HMT,* Homology mediated translocations; *OMT,* off target mediated translocations.

**Fig E5:**
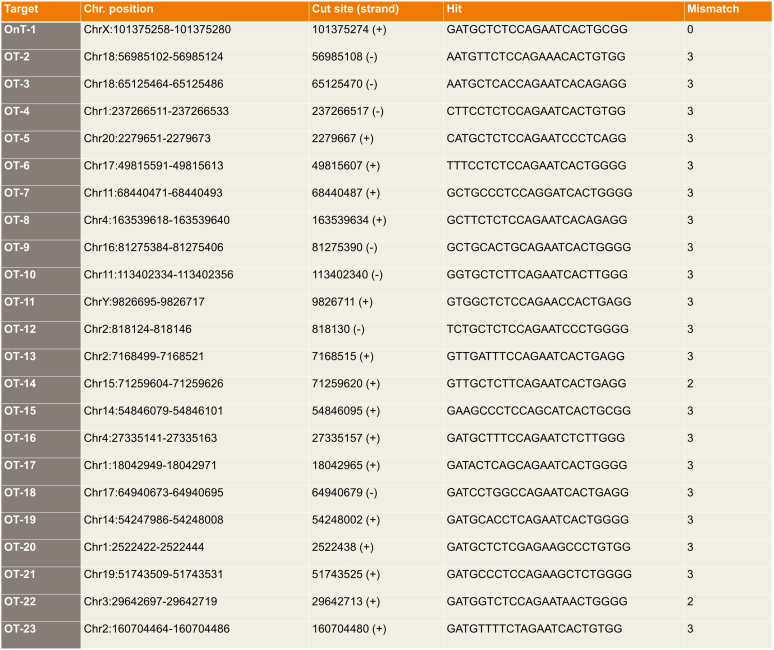
Predicted off-target sites. List of 23 target sites: 1 on-target (On-T) and the top 22 off-target (OT) sites predicted by the COSMID online tool when inputting the sequence of our chosen gRNA and allowing for up to 3 mismatches.

The introduction of undesired genetic alterations and the possibility of significant chromosomal rearrangements are potential concerns of many gene editing approaches. To assess the precision of our chosen gRNA, we electroporated the high-fidelity CRISPR/Cas9 complexed with *BTK* gRNA into 2 XLA HSPCs and examined the presence of INDELs at the top 22 off-target sites that were predicted by the bioinformatic tool COSMID allowing for up to 3 mismatches to the on-target sequence (see Fig E5 in this article’s Online Repository at www.jacionline.org).^26^ By performing deep sequencing, with a read depth of 50,000×, of both Cas9/gRNA RNP-electroporated and untreated XLA HSPCs, we did not find substantial genetic disturbances occurring at a frequency >1% in edited versus control samples (Fig 6, *A*). Two off-target sites exhibited INDELs at a significantly higher frequency in the edited sample, although still at <1%. Of note, both sites were located within intronic regions; one was within intron 6 of the thrombospondin type 1 domain locus on chromosome 15 (off-target site 14) and the other within intron 21 of the low-density lipoprotein receptor-related protein 5 locus on chromosome 11 (off-target site 7). Both off-target sites were previously identified using a genome-wide, unbiased identification of DSBs enabled by sequencing^40^ approach in cells edited with WT CRISPR/Cas9, but again these sites were deemed negligible when a high-fidelity CRISPR/Cas9 was employed.^20^ We analyzed the same samples using CAST-seq,^25^ an unbiased methodology that detects gross chromosomal abnormalities, which revealed the presence of anticipated large deletions near the on-target site, at a frequency of about 2% of the alleles (Fig 6, *B*). Interestingly, we also detected additional mutations at extremely rare frequencies (<0.001% of the alleles) in only 1 HSPC donor source: one on chromosome 18 that does not seem to be associated with CRISPR/Cas9 off-targeting activity, and the other on chromosome X, possibly induced by the presence of homologous sequences driving the DNA repair process.

## Discussion

Advancements in gene editing technologies are rapidly resulting in the development of new cell-based therapies for inborn errors of immunity.^41^ Here we show the successful application of a gene editing strategy to rescue BTK expression and B-cell differentiation in HSPCs from patients with XLA using an optimal HDR donor cassette (Fig 1).

Targeted integration of a BTK coding sequence in its native locus ensures that expression remains under the control of physiological regulatory elements, reducing the risks associated with deregulated expression and genotoxicity observed in retroviral gene therapy approaches, where copies are integrated semirandomly into the patient's genome.^42^ It is indeed important to note that the levels of BTK protein obtained in the DG75 B-cell clones after targeted integration of our optimal donor cassette are comparable to those of WT cells and that there is no ectopic expression in Jurkat T cells, reflecting a physiological pattern of BTK expression.

Using our protocol, we could reliably obtain targeted transgene insertion in >30% of human HSPCs, when employing either AAV6-GFP or our therapeutic AAV6–co-BTK vectors as donor templates. The AAV6-GFP vector was also used to create BTK KO primary cells to optimize B-cell differentiation protocols sparing precious patients’ material. *In vitro* B-cell differentiation from CD34^+^ cells has been generally accomplished through their coculture with supporting feeder cells^43^, ^44^, ^45^ or by adding supernatants derived from mesenchymal stem cell cultures.^46^ While these *in vitro* methodologies have proved effective to some extent, the use of poorly characterized feeder cells and the rapid phenotypic changes of feeder cells with passaging makes reproducibility an issue. Here we adopted a feeder-free system^33^ that relies on the use of IL-6 subsequently replaced by IL-7 to drive the expansion of early precursors. Subsequently, there is an IL-7–independent stage when on removal of IL-7, immature B cells emerge.^47^^,^^48^ A clear significant difference in the end product (immature B cells) could be established between XLA and WT samples, allowing us to study the efficacy of gene editing with this protocol. Of note, a similar pattern of B-cell differentiation was obtained when using nonmobilized CD34^+^ cells (data not shown), warranting its further use to model and study therapies of B-cell disorders. Indeed, by the end of the culture period, we could show the appearance of cells expressing CD10^+^/CD19^+^/IgM^+^ in WT samples, absent in cultures from patients with XLA and rescued on gene editing. To confirm that the cells rescued with gene editing can further mature into antibody-producing B cells, we used coculture systems with T_H_ cells in the presence of SEB. SEB facilitates T-cell receptor interaction with MHC class II molecules on immature B cells in a nonspecific manner^49^ so that the CD40 ligand expressed on activated CD4^+^ cells binds to CD40 on B cells,^50^ resulting in T-cell activation and B-cell class switching from IgM to IgG.^51^ In this system we showed rescued T-cell activation and B-cell class switching on gene editing.

Ultimately, it was important to evaluate the ability of gene edited XLA HSPCs to engraft and rescue the XLA phenotype *in vivo*, and we could establish that gene edited cells were able to engraft although to a lesser extent than nonedited cells. It is well reported that the gene editing procedure and suboptimal AAV6 purity causes cell cycle arrest and toxicity that could be partially ameliorated using p53 inhibitors, clinical grade reagents, and alternative HDR donors.^37^^,^^52^, ^53^, ^54^ Our experience, in line with others,^54^^,^^55^ shows that using lower AAV MOIs in combination with optimal combinations of gRNA and Cas9 can significantly improve the fitness of corrected cells.^56^

One limitation of this model is that the XLA phenotype is not fully recapitulated.^39^ In patients with XLA, scattered nonfunctional peripheral B cells may be found. This phenomenon is exacerbated in xenotransplant model systems. Indeed, while none of our XLA donors had circulating peripheral B cells, their HSPCs gave rise to a CD19^+^ population in the blood of transplanted animals. This is very similar to what is observed in X-linked immunodeficient mice with mutations of *Btk.*^57^ The murine phenotype is partly due to the function of the murine Tec protein, but one cannot exclude the involvement of the murine cytokine milieu and stroma. Instead of a complete block in B-cell maturation, we observed a lower frequency of the CD19^+^/CD34^−^ B-cell population in the bone marrow of mice transplanted with HSPCs from patients with XLA if compared with WT HSPCs transplanted mice with gene editing reverting this phenotype. CD19^+^ cells, although present in the blood of XLA mice, were at a lower frequency than those in WT and edited samples, and they were unable to produce significant levels of IgM and IgG. More importantly, gene editing rescued both IgM and IgG production and resulted in the appearance of KRECs, a by-product of the immunoglobulin heavy and light chain rearrangement.^58^ Therefore, we have established here that the detection of KRECS by ddPCR, conventionally used as screening test for congenital agammaglobulinemia,^59^ can also be employed in mouse models to assess the efficacy of cell therapies aiming to restore B-cell development. Indeed, the deleterious effects of cell manipulation on the engraftment of human cells and the absence of a severe phenotype in transplanted mice might have overshadowed the potential selective advantage that corrected cells should have over XLA cells.

Another limitation of this system is that B cells do not reach full maturation. Alternative systems might be preferable to study the humoral response to vaccination, such as a humanized mouse model^60^ on a *Btk/Tec*^−/−^^61^ background.

For clinical translation, it is essential to evaluate safety. By next-generation sequencing analysis, we confirmed editing at 2 previously reported off-target sites located within the intronic regions of genes,^20^ although at extremely low levels (<1%). Of note, using the CAST-seq methodology, no evident large chromosomal rearrangements between the on-target and the 2 off-target sites were found, suggesting that the designed gRNA targeting *BTK* is a relatively safe choice. Novel Cas systems targeting the same locus with improved specificity^15^^,^^62^ could also be evaluated. Overall, the CAST-seq unbiased methodology found translocation events at very low frequencies (<0.001%) mainly involving naturally occurring breaking sites. As we used unmanipulated cells rather than mock electroporated cells as experimental controls, we cannot exclude the possibility of the electroporation procedure itself influencing DNA nicking. Importantly, the observed translocations and their nearby genes within 100 kb had not been previously associated with oncogenic potential, suggesting they do not pose a significant genotoxic risk.

In conclusion, we have successfully shown in different systems that our gene editing strategy rescues the XLA phenotype and consistently relieves the B-cell differentiation block in a significant proportion of XLA HSPCs both *in vitro* and *in vivo*. Considering the potential selective advantage of corrected cells expected when operating in the disease setting, we believe this strategy could provide significant clinical benefit for patients with XLA.

## Disclosure statement

The work was supported by the 10.13039/501100000265Medical Research Council, United Kingdom (SB-MR/S021930/1) and the 10.13039/100010269Wellcome Trust, United Kingdom (217112/Z/19/Z). A.J.T., C.B., A.C., and G.S. were also supported by the National Institute for Health and Care Research Biomedical Research Centre at 10.13039/501100003784Great Ormond Street Hospital for Children National Health Service Foundation Trust and 10.13039/501100000765University College London. E.B. was supported by a postdoctoral grant from Fundación Alfonso Martín Escudero, Spain.

Disclosure of potential conflict of interest: A. J. Thrasher is on the Scientific Advisory Board of Orchard Therapeutics, Generation Bio, Carbon Biosciences, and 4BIO Capital. E. C. Morris is a founder shareholder of Quell Therapeutics Ltd and has received honoraria from Orchard Therapeutics, GlaxoSmithKline, and AstraZeneca. C. Booth has performed ad hoc consulting in the past 3 years for SOBI and Novartis and educational material production for SOBI and Chiesi. E.G.C. is a current employee and shareholder of GlaxoSmithKline. The rest of the authors declare that they have no relevant conflicts of interest.

**Key messages**

• XLA is an inborn error of immunity that results in the absence of mature B cells. Current management centers on life-long immunoglobulin replacement therapy.
• We describe a universal gene editing strategy in HSPCs from patients with XLA that restores B-cell development and immunoglobulin production.
• This represents a promising and potentially curative future treatment for XLA.

## Figures and Tables

**Table E1:** Primers and probes

Target	Forward primer	Reverse primer	Probe
Co-BTK integration	CCAGTGGTGACTTTGGGAACT (intron 1)	GTCGATGCTGCCTTTCTTGC (co-*BTK*)	FAM CTGGAGTATTGTAGGTTTGGGGAGGCT
Albumin	GCTGTCATCTCTTGTGGGCTG	CACAAATTTGGAAACAGAACAGGCATT	HEX CCTGTCATGCCCACACAAATCTCTCC
KREC	TCCCTTAGTGGCATTATTTGTATCACT	AGGAGCCAGCTCTTACCCTAGAGT	HEX TCTGCACGGGCAGCAGGTTGG
TRAC	TGGCCTAACCCTGATCCTCTT	GGATTTAGAGTCTCTCAGCTGGTACAC	FAM TCCCACAGATATCCAGAACCCTGACCC
Exon 2 (*BTK*) Cut locus	AGGTGCGTGCCATTTAACATCTA	ACCTTTGACTTTCCGGCTTTAGC	N/A
Co-BTK (cDNA)	GAGTCCAACCTGCCCTGGTGG	GATCAGCTCGGGGATGGTGCTG	SYBR green
WT BTK (cDNA)	CACTTGTGTTGAAACAGTGGTT	TCAGGCGTTGGGGGAAGAGG	SYBR green
GAPDH (cDNA)	CCAAGGAGTAAGACCCCTGG	TGGTTGAGCACAGGGTACTT	SYBR green
OnT-1	TGAAACTAGGTAGCTAGGCTGAGG	CATAGTCGAGAAACTTACCCCACG	N/A
OT-2	CGACTGCCTGTGGCTCTTGA	GTTGACAATATATGCAAAGCTAGCACTGGC	N/A
OT-3	CAGGAACACTAGCCAAGGGTAAG	GCCAAAAGCTCTCAGCCAACATC	N/A
OT-4	GAGCATGTGTGCATGTACACAATATCCTCA	GCCCAGGAGCTGCTTCTATTTTAC	N/A
OT-5	CCCATGTGCTGGGTTGTCAAG	CTGCATTCAGGAGACTCAAAACCC	N/A
OT-6	CAATCCATGCAAACCACACAACCATAAGC	CTGGGAATTTGTAGTGTCTCTGGG	N/A
OT-7	CAGACAAACACAGGGTGATTTT	CTCACCTTAGGGGCATACAGCTGC	N/A
OT-8	TAAGAGCGGGGCTTTCACAGG	CTGTGGTCTGAAAGGTTGGCAGAA	N/A
OT-9	CTGGCTGCACATTGGGATCC	CTTAGGCTTGAACAGGAAGAGGG	N/A
OT-10	AGCCTGCACGTTAAAATCTCCTGG	CGTCAGGAGTCCAAATGGAAGAG	N/A
OT-11	TCCTACTACCACACACTCTCAAAGGATTTC	GGCATTGTCTGGTTTCAGTAGCCT	N/A
OT-12	TCGGCCTTTCAGACTCTTCTGAGC	GGACACACACTAATGCATGCATGG	N/A
OT-13	GAACAGCTCAAGTATAACAAGGTACCTAGG	CTGGAAGTAACTAATGCATCCAAGCTGATC	N/A
OT-14	TGCTCCAGCTGATAGTAGCAGATG	TGAGAGGTTCTCTACCCTAGCTAC	N/A
OT-15	GAGCCAAGACACACCAGCTTTTAC	GACTGCAGCCCCTTTGTTAACC	N/A
OT-16	AACCCCTAATGAGTATTGCTGAGAGGTTG	ATCCCAGCCTTGTGATACCCG	N/A
OT-17	AATTGTGGCAGGCAGGGTAAAGAG	CCACCACCTCATCAGGCCAT	N/A
OT-18	CAGATGAACAGCAGCAGCAAAGAG	AGCTGACTCTGGCCATACCAC	N/A
OT-19	ACCCACCTTGGTCTCCCAAAGT	GGAGAAGTCCCCAAATGTCATCTG	N/A
OT-20	GGCCTTCTTCAAGAATCTTTTCACTTTTCCC	AGCTCCCCTACCCTGGAGA	N/A
OT-21	GATGTGGTCCTTGGGAATCTAGC	GACCAAAGGGGTTCCAGGATTTAC	N/A
OT-22	GGGACAGCAGAACAAGACTGTG	GACTGAGGGAAAGGCAGACTTG	N/A
OT-23	CCAGTTGGTGTCCTCTTTCTGC	CCCCACCAAGAGAAGAACCTAAG	N/A

*N/A*, Not aapplicable; *OnT*, on-target site; *OT*, off-target site.

**Table E2:** -ddPCR amplification conditions

Target	KRECs, TRAC	Co-BTK, albumin
Preincubation	15 min, 95°C	10 min, 95°C
Cycle: denaturation	30 s, 95°C (ramp 2.5°C/s)	60 s, 95°C (ramp 1°C/s)
Cycle: annealing Cycle: extension	60 s, 60°C (ramp 2.5°C/s)	30 s, 60°C (ramp 1°C/s) 6 min, 72°C (ramp 1°C/s)
Cycles	50	50
Inactivation	10 min, 95°C	10 min, 95°C

## Methods

### AAV6 production

We mixed 165 μg of the donor template and 484 μg the pDGM6 helper plasmid with polyethyleneimine (cat no: 23966, Polysciences, Warrington, Pa). This mixture was used to transfect 330 million HEK293T cells cultured in 22 150-mm diameter culture dishes with Dulbecco modified Eagle medium supplemented with 2% FCS and 1% penicillin streptomycin. After 72 hours, virus particles in the pooled supernatants were precipitated with ammonium sulphate (31.3 g/100 mL). This precipitate was isolated with centrifugation at 8300*g* for 30 minutes before being resuspended in 10 mL of PBS with 2.5 mmol/L potassium chloride and 1 mmol/L magnesium chloride. This was then incubated with Benzonase nuclease (50 U/mL) (cat no: E1014-5KU; Merck, Kenilworth, NJ) for 30 minutes at 37°C. Separately, the cells were scraped, resuspended in 10 mL of PBS and freeze-thawed for 3 cycles. Then 10% deoxycholic acid was added to make a final concentration of 0.5% and the solution was incubated at 37°C for 30 minutes with Benzonase nuclease (50 U/mL). The solution was centrifuged at 4000*g* for 30 minutes, and the supernatant was combined with the virus from the supernatant.

This was ultracentrifuged at 273,800*g* for 3 hours at 18°C in ultracentrifuge tubes containing 15%, 25%, 40%, and 60% gradients of iodixanol using a Beckman Coulter (Pasadena, Calif) Optima XPN-100. The resulting layer between the 40% and 60% gradients, which contained virus particles, was extracted and purified using the Slide-A-Lyzer Dialysis Cassette kit 10,000 MWCO (Thermo Fisher Scientific) with 2 2-hour PBS cycles and 1 cycle in 5% D-sorbitol solution. The QuickTitre AAV Quantitation Kit (cat no: VPK-145; Cell Biolabs, Inc, San Diego, Calif) was used to quantify the virus. Titers were expressed as vector genomes/mL.

### Detection of BTK by Western blot

From 2 million to 4 million cells for cell lines and 500,000 million for primary cells were washed with PBS and incubated on ice for 15 minutes with 50 μL of radioimmunoprecipitation assay buffer (Sigma-Aldrich, St Louis, Mo) containing cOmplete, Mini Protease Inhibitor Cocktail (cat no: 11836170001; Roche Diagnostics, Indianapolis, Ind). Then 25 μg of protein from each sample was run in a 4% to 12% bis-tris protein gel. A wet transfer method was used to transfer proteins to a Millipore Immobilon-P membrane (MilliporeSigma, Burlington, Mass). That membrane was probed with anti-BTK (1:2000; cat no: MAB5807; R&D Systems, Minneapolis, Minn) and anti-GAPDH (6C5) (1:1000; cat no: sc-32233; Santa Cruz Biotechnology, Dallas, Tex) antibodies, respectively. Bands were revealed using the SuperSignal West Pico PLUS Chemiluminescent Substrate kit (cat no: 34580, Thermo Fisher Scientific) and visualized with the ChemiDoc MP Imaging System (Bio-Rad).

### INDELs quantification

INDEL frequencies were quantified using an amplicon spanning the DSB site in exon 2 with the TIDE software (http://shinyapps.datacurators.nl/tide/). Primers are detailed in Table E1.

### Separation of CD34^+^ cells from apheresis samples using CliniMACS Prodigy platform

CD34^+^ separation from 3 patients with XLA was completed using the CliniMACS Prodigy Platform (Miltenyi Biotec). The TS310 tubing set (Miltenyi Biotec) was installed following the on-screen instructions, and the recommended integrity test was completed. One vial of ClinicMACS CD34 Reagent (Miltenyi Biotec) was used per 600 million cells. Selection buffer (comprising 75 mL of Zenalb 20% human albumin solution [Bio Products Laboratory Limited, Elstree, United Kingdom]) was added to each of 2 3-L bags of CliniMACS PBS/EDTA Buffer (Miltenyi Biotec). Elution buffer and 5% IVIG were connected. The apheresis program LP34 was then run. The purified CD34^+^ cells were counted, washed, and resuspended in CryoStor CS5 (STEMCELL Technologies) media. They were frozen in 1.0-mL Cryovials (cat no: 377224, Thermo Fisher Scientific) containing 1 million, 2 million, 5 million, 10 million, or 20 million cells. Healthy donors CD34^+^ cells were separated manually using the CD34 Microbead Kit (Miltenyi Biotech) following manufacturer’s instructions.
